# Adaptive Neuro-Fuzzy-Based Models for Predicting the Tribological Properties of 3D-Printed PLA Green Composites Used for Biomedical Applications

**DOI:** 10.3390/polym15143053

**Published:** 2023-07-15

**Authors:** Thamer Albahkali, Hany S. Abdo, Omar Salah, Ahmed Fouly

**Affiliations:** 1 Mechanical Engineering Department, College of Engineering, King Saud University, Riyadh 11421, Saudi Arabia; talbahkali@ksu.edu.sa; 2 The King Salman Center for Disability Research, Riyadh 11421, Saudi Arabia; 3 Center of Excellence for Research in Engineering Materials (CEREM), Deanship of Scientific Research, King Saud University, Riyadh 11421, Saudi Arabia; habdo@ksu.edu.sa; 4 Mechanical Design and Materials Department, Faculty of Energy Engineering, Aswan University, Aswan 81521, Egypt; 5 Department of Mechatronics, Faculty of Engineering, Assuit University, Assuit 71515, Egypt; omar.salah@aun.edu.eg; 6 Department of Production Engineering and Mechanical Design, Faculty of Engineering, Minia University, Minia 61519, Egypt

**Keywords:** PLA green composite, 3D printing, ANFIS, artificial intelligence, rehabilitation medicine

## Abstract

Tribological performance is a critical aspect of materials used in biomedical applications, as it can directly impact the comfort and functionality of devices for individuals with disabilities. Polylactic Acid (PLA) is a widely used 3D-printed material in this field, but its mechanical and tribological properties can be limiting. This study focuses on the development of an artificial intelligence model using ANFIS to predict the wear volume of PLA composites under various conditions. The model was built on data gathered from tribological experiments involving PLA green composites with different weight fractions of date particles. These samples were annealed for different durations to eliminate residual stresses from 3D printing and then subjected to tribological tests under varying normal loads and sliding distances. Mechanical properties and finite element models were also analyzed to better understand the tribological results and evaluate the load-carrying capacity of the PLA composites. The ANFIS model demonstrated excellent compatibility and robustness in predicting wear volume, with an average percentage error of less than 0.01% compared to experimental results. This study highlights the potential of heat-treated PLA green composites for improved tribological performance in biomedical applications.

## 1. Introduction

Age, being overweight, and accidents are the main reasons for increasing osteoarthritis [1]. One of the most affected human joints that can cause disability is the knee joint [2]. The most effective candidate for the treatment of knee joint osteoarthritis is replacing the affected joint with an artificial one [3]. This treatment has been proven to improve the quality of life for those with knee joint disabilities, and it is expected to continue as a leading treatment in the future [4]. Developing reliable and durable artificial knee joints is crucial to avoid postoperative instability and the need for maintenance and revision surgeries. Biomedical scientists are working to create artificial knee joints from materials with appropriate properties. However, these materials require further improvement in their mechanical and tribological properties to withstand various loading conditions. This can be achieved by exploring and developing new materials and new manufacturing techniques and utilizing advanced simulation and characterization tools to optimize the performance of the artificial knee joint materials.

Although ceramic materials have been widely considered the most suitable for knee joints due to their excellent tribological properties, their brittleness is a significant drawback to their use [5]. Studies have shown that the brittleness of ceramics can result in 2% of joint fractures [6] as well as in the squeaking of synthetic ceramic prostheses [7]. These disadvantages have prompted researchers to consider using metals with sufficient mechanical properties and flexible design options [8]. Despite the potential benefits of bioactive metals like titanium and their alloys in binding to bones spontaneously, their use in artificial joints is limited by the potential for corrosion and wear [9]. Studies have shown that corrosion and wear in metallic joints can negatively impact their durability and reliability [10]. The metal debris and ions that leach from the joint can lead to inflammation and necrotic tissue surrounding the metallic joint [11,12,13].

Polymers have become a focal point for researchers in recent times owing to their distinct benefits. These long molecular chains are versatile and can be easily synthesized to create custom materials with specific properties, making them highly adaptable. Polymers are also lightweight, flexible, and inexpensive, making them ideal for a range of applications in industries such as healthcare, electronics, and packaging. Additionally, polymers are considered sustainable and eco-friendly, and their ability to reduce waste, lower costs, and increase energy efficiency makes them a popular choice across various industries [14,15,16]. Biomedical polymers, also known as biopolymers, are characterized by their biostability and biodegradability properties, making them suitable for use in various biomedical applications. These materials can resist breakdown in the body, ensuring long-term durability while also being able to degrade and be absorbed by the body, minimizing the risk of harmful effects. Biopolymers can be used for a variety of applications, including drug delivery systems, tissue engineering, and medical devices [17]. The development of new biopolymers is an evolving field with the potential to impact the future of biomedical technology [18,19,20]. The biomedical use of polymers began with a PMMA intraocular lens created by a British ophthalmologist. This invention marked a milestone in the field of medicine, and since then, polymers have been extensively used in diverse biomedical applications, such as sutures, artificial heart valves, and drug delivery systems [21,22].

Additive manufacturing, which involves using 3D printers to create intricate 3D structures, has become increasingly popular in biomedical research. In particular, researchers have utilized this technique to develop complex biomedical parts in fields such as oral and maxillofacial surgery. The versatility of 3D printers allows for the creation of highly customized and precise parts that can improve patient outcomes and reduce surgical complications. Additive manufacturing continues to be an attractive option for researchers in the biomedical field looking to develop innovative solutions to complex medical problems [23,24]. Among the many polymers that can be used in 3D printing, Polylactic Acid (PLA) stands out as a popular choice because of its low warping issues, environmentally-friendly nature, biocompatibility, and ease of use. PLA is a versatile and cost-effective option for 3D printing enthusiasts and professionals alike. Due to its eco-friendly properties, it is also a popular choice for creating sustainable products and prototypes. When it comes to 3D printing, PLA remains a top contender in terms of quality, reliability, and versatility [25]. However, its practical applications are limited because of its low stiffness and strength [26]. To address the limitations of PLA, researchers have tried blending it with other polymers, adding fillers, and using advanced processing techniques. This aims to enhance its properties, such as thermal stability, mechanical strength, and moisture resistance. Blending with other polymers such as ABS, PET, and PC can improve heat resistance and toughness. Fillers like carbon nanotubes, clay, and cellulose can enhance mechanical strength while maintaining their biocompatibility and environmental friendliness. Advanced processing techniques such as extrusion, injection molding, and 3D printing can also improve PLA’s performance for various applications. These improvements could make PLA a more viable alternative to traditional polymers [27].

Natural fillers such as wood flour, starch, and cellulose have been found to be effective as a reinforcement to polylactic acid (PLA). These fillers can improve the mechanical properties of PLA, such as stiffness and strength, while also reducing costs and environmental impact. Wood flour, for example, has been shown to greatly improve the toughness of PLA-based composites, making them suitable for structural applications. Furthermore, the use of natural fillers in PLA composites can also contribute to the development of sustainable and biodegradable materials [28]. Liu et al. [29] investigated the effects of adding natural materials—eucalyptus, lignin, pine, and pulp—to PLA at different concentrations. The study looked at the print quality, melt flow index, and mechanical properties of the resulting composites. The team discovered that incorporating natural materials improved the composite’s tensile strength by more than 70% when using 15% lignin additives. An additional study was conducted by Fouly et al. [30], who investigated the impact of reinforcing 3D-printed PLA with date pit filler at different concentrations. They found that the mechanical properties of the PLA composites slightly improved without any modification or additional process due to the inclusion of date pit filler up to 10 wt.%. The research highlights the potential of using sustainable fillers to enhance the mechanical properties of 3D-printed parts. Previous research indicates that 3D-printed products tend to exhibit suboptimal mechanical and tribological (friction and wear) properties, regardless of the material employed in the 3D printing process. This suggests that additional measures may be necessary to improve the quality and durability of 3D-printed objects, particularly in applications where high mechanical strength and wear resistance are required [31].

Heat treatment is regularly used in the metal industry to modify the properties of metallic materials and to lessen residual stresses created during fabrication. Similarly, internal stresses occur during the 3D printing process, and heat treatment is utilized to alleviate these stresses [32]. Scientists suggest that the heat treatment process could be used to alter the internal structure of polymers as well and improve their strength, durability and other characteristics. However, further research is needed to determine the optimal conditions for heat-treating polymers and to test the durability of treated materials over time [33]. Bhandari et al. [32] conducted research on a PLA–carbon composite and found that heat treatment led to a significant improvement in the composite’s mechanical properties compared to pure PLA. The study suggests that heat treatment could be a viable method of enhancing the performance of PLA–carbon composites, which have potential applications in a wide range of industries, including aerospace and automotive manufacturing. Annealing 3D-printed polylactic acid (PLA) parts at 100 °C for improved tensile properties and heat resistance has been demonstrated in a study by Jayanth et al. [34]. Samples exposed to temperatures of 0, 90, 100, and 120 °C showed an 80% increase in tensile properties when annealed at 100 °C, with a corresponding 73% improvement in heat resistance. This finding highlights the potential for improving the mechanical performance of PLA parts through post-processing treatments.

Models have been developed to establish the relationship between specific parameters that affect the quality and properties of 3D-printed products and their performance. These models provide a valuable tool in optimizing the 3D printing process, as well as predicting the behavior of the printed parts. By using these models, manufacturers can ensure that their products meet strict quality standards while minimizing production costs and time. As technology develops, these models are expected to become increasingly sophisticated and accurate [35,36]. Sahin et al. [37] studied the wear rate of aluminum metal matrix composites concerning sliding distance, the weight fraction of additives, the size of additives, and applied load. Using statistical methods, they established a relationship between the wear rate (output) and input parameters. Other researchers have also used the Taguchi method to develop a relationship between inputs and wear rate [38]. ANFIS is an advanced tool that combines the power of fuzzy systems and neural networks to estimate input–output relationships in complicated, non-linear scenarios [39]. It is widely regarded as a superior option for solving complex problems where conventional statistical techniques do not provide satisfactory results. ANFIS can be used in a variety of applications in various domains, such as finance, medicine, manufacturing, and engineering, to name a few. With its ability to learn and adjust its parameters based on incoming data, ANFIS can produce accurate predictions and make it an essential tool for any data analyst or scientist working in complex environments. Chhabra et al. [40] looked into how different parameters of 3D printing impact carbon-reinforced polyamide composite. They employed a combination of genetic algorithm and ANFIS, called GA-ANFIS, to anticipate how well the 3D-printed composite would perform in terms of wear based on the printing parameters used. Ibrahim et al. [41] evaluated the wear rate of polytetrafluoroethylene composites through different factors, including reinforcement weight fraction, composite density, and sliding distance, using three prediction models. They compared the multilinear regression model’s performance with an artificial neural network and an adaptive network-based fuzzy inference system and found that the multilinear regression model outperformed the others with an accuracy of 97.4%. This study’s findings can be used to optimize the wear resistance of PTFE composites for various applications in the automotive, aerospace, and biomedical industries.

The current study uses ANFIS to develop an artificial intelligence model that estimates the tribology characteristics of PLA green composites in liner spacers used in artificial knee joints as a biomedical application. Polylactic acid (PLA) was mixed with different percentages of date pit particles, and annealing at 100 °C was conducted on the samples for varying durations to reduce residual stress and prevent voids formation. The weight percentages of date pit particles used ranged from 0% to 10% with a 2% step. The mechanical and wear resistance properties of unannealed and annealed PLA–DP composites were assessed, while a FEM model was created with ANSYS to mimic the conditions that would be present in wear testing, providing further insight into the performance of the composites under different conditions. In conclusion, ANFIS models were created through MATLAB that accurately predicted the wear performance of PLA composites based on the weight fraction of DP, annealing time, normal load, and sliding distance. These models can be extremely beneficial to different industries requiring a better wear-resistant material, as PLA composites with DP have proven to be a promising candidate.

## 2. Materials and Experimental Work

### 2.1. Sample Preparation

In this study, PLA in filament form was purchased from Shanghai Nuolei CNC Router Equipment Co., Ltd., Shanghai, China, and date pits were produced from Khoas dates, which are available in the local market. The composition of Kholas dates is 67.6–74.2% dietary fiber, 5.7–8.8% lipids, 4.8–6.9% protein, 8.6–12.5% moisture, 2.4–4.7% carbohydrates and 0.8–1.1% ash [42]. The process of producing date pits is started by hard washing to remove any contaminations prior to grinding and obtaining the green powder. The date pit powder was then dried for one week using a drying oven (JSV0–60T) at 60 °C to remove all moisture. In order to reduce the date pits’ particle sizes, fine grinding using a ball milling machine was carried out at 200 rpm for 8 h with 15 min intervals. Then the morphology of the produced date pit powder was analyzed using a scanning electron microscope.

**Figure 1:**
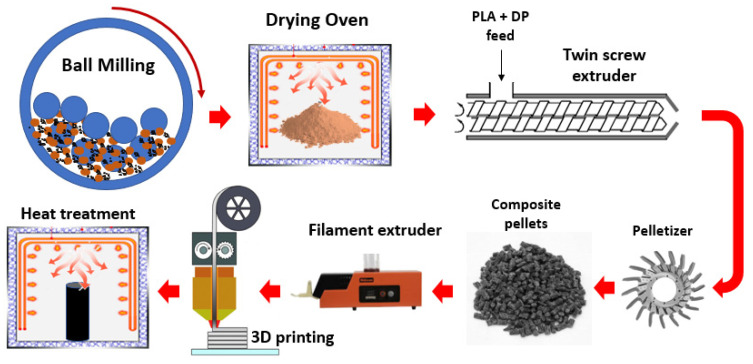
The detailed preparation process of PLA–DP composites; starts from preparing the date pit powder until the heat treatment process for the composite sample.

PLA filament was converted in pellet form through a pelletizer in order to mix with different DP percentages (2, 4, 6, 8 and 10 wt.%) using a twin screw extruder with a tip temperature of 210 °C. As shown in the schematic diagram (Figure 1), the output of the twin extruder was feeding the pelletizer to produce small pellets of PLA/DP composite. The final step in the production process is 3D printing and sample fabrication. For that purpose, a Creality 3D Ender-3 V2 3D printer was used to produce 100% density samples from the filament produced through a Filabot EX2 single-screw extruder. The 3D printer settings were adjusted to 200 to 210 °C nozzle temperature and 70 °C the bed temperature. The heat treatment process was carried out on the produced samples at 100 °C and for a different time duration of 0, 2.5, 5, 10, and 20 h. The sample designations are illustrated in Table 1a,b.

### 2.2. Characterization and Testing

According to ASTM D2240, the hardness of the produced composite samples was measured via Shore D durometer [43], while samples were compressed according to the ISO 604 [44] using an Instron 5582 testing machine and a strain rate of two mm per minute, in order to evaluate the mechanical characteristics needed for the simulation part. The wear volume and friction coefficient of PLA–DP composite samples were specified in dry conditions using the pin-on-disc test, according to ASTM G99-95 [45], universal tribometer Mod. UMT-2MT testing block sin T45815 Bruker-Nano Surfaces. The tribological tests were conducted at a speed of 0.4 m/s, temperature of 26 °C, and humidity of 60%. The PLA–DP composite sample dimensions are 20 mm in length and 8 mm in diameter. The disc is stainless steel with a diameter of 8 cm and 12.5 µm surface roughness. After each experiment, the stainless-steel plate was cleaned using acetone and dried with a heat gun to avoid impurities from previous experiments. The tribological outcomes were recorded under different normal forces and sliding distances. The procedure was conducted six times for each specimen, and the mean friction coefficient was computed. Each specimen’s weight was measured before and after the tribological test, and the wear volume was computed. To investigate the wear mechanism after the frictional test, the authors scanned the rubbed surfaces using a scanning electron microscope (SEM), and based on analyzing the images, the wear mechanisms were identified.

### 2.3. Finite Element Analysis

**Figure 2:**
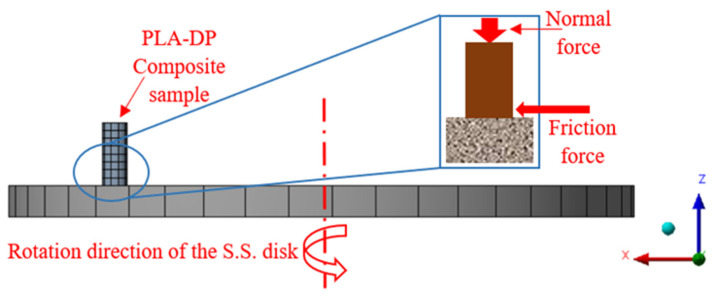
Simulation models of the frictional process.

By studying the distribution of contact stresses, the load-carrying capability of materials can be determined [46]. As a result, stress on contact must be measured during these procedures to establish the load-carrying capacity of the PLA–DP composites in response to the friction process. Due to the complexity of measuring the stresses generated due to friction on the knee joint model, a simulation was created to represent the various stresses that occur during the friction process between the pin and disk, as illustrated in Figure 2. The contact between the pin and disk was modeled as frictional, and the solution was based on the Lagrange contact method. The friction coefficient was defined based on the experimental results. The disk material is set as stainless steel, and the pin is selected based on PLA–DP composites. The model is meshed as a combination of hexahedrons and tetrahedrons, with 325 elements and 2260 nodes. The PLA–DP sample was subjected to a 30 N normal load in the *z*-direction but fixed in the other directions.

### 2.4. Adaptive Neuro-Fuzzy Inference System (ANFIS)

**Figure 3:**
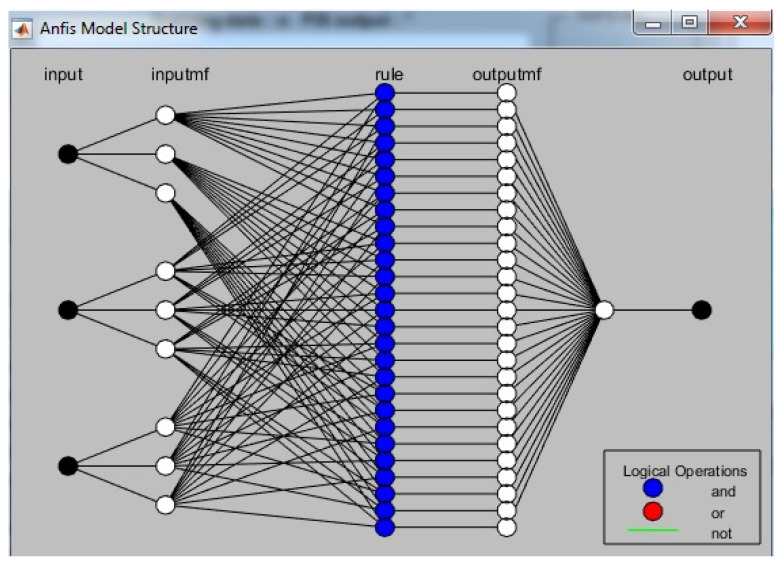
ANFIS architecture.

The ANFIS model is a combination of fuzzy logic and artificial neural networks. While fuzzy logic is useful in converting qualitative inputs to clear outputs, it lacks a specific conversion method and may take a longer time to adjust membership functions. In contrast, artificial neural networks have a strong ability to learn from data and can quickly adjust to changing inputs. By combining the two, ANFIS models can create more effective and accurate models for a variety of applications. The ANFIS structure used in this study consists of three inputs and one output. The inputs are DP weight fraction, heat treatment duration time, and normal load or sliding distance. The output is wear volume. This structure allows for the development of a model that can accurately predict wear volume based on these three input parameters. Ultimately, this can help in better understanding the factors that contribute to wear in DP composites and can guide material designers in the proper selection for various engineering applications. As shown in Figure 3, the ANFIS structure used in this study can be visualized. The ANFIS structure consists of three inputs (in the current study, DP weight fraction, heat treatment duration time, and normal load or sliding distance) and one output (wear volume). The figure shows the use of three membership functions for each input.

ANFIS, or Adaptive Neuro-Fuzzy Inference System, is a five-layered network structure that combines fuzzy logic and neural networks to make predictions based on input data. The first layer, called fuzzification, transforms input variables into linguistic values using membership functions. The second layer, called the product layer, multiplies these linguistic values and sends the result to the third layer, called the normalization layer. The fourth layer called the rule layer, combines rules from the previous layers to make inferences. Finally, the fifth layer, called the defuzzification layer, converts these inferences back into numerical values to output a prediction.

The ANFIS network is a powerful tool that can adaptively map inputs to their corresponding outputs by using a combination of membership functions, rule bases, and parameters. This is achieved through a supervised learning process that trains the network using a dataset of input–output pairs. Once trained, the ANFIS network can be used to make accurate predictions and classification decisions based on new input data. Overall, ANFIS is a valuable approach for building intelligent systems that can simulate and enhance human decision-making.

## 3. Results and Discussion

**Figure 4:**
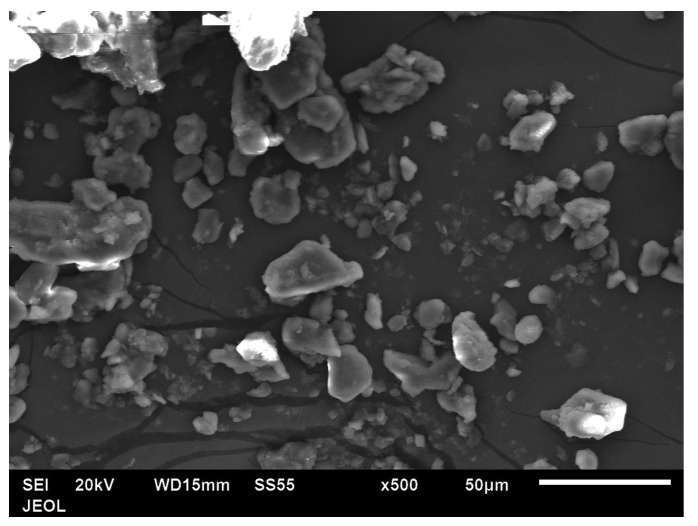
The morphology of date pit SEM after 8 h of ball milling.

The preparation of date pit powder is started by the grinding process involving multiple stages. Figure 4 illustrates the morphology of the date pits particles after the preparation process. The morphology of date pit particles is shown after eight hours of ball milling, and the particle shapes are flaky.

Table 2 presents the Shore-D hardness values with variations in the weight fraction of date pits and heat treatment duration time. The results indicate that increasing the weight fraction of date pits results in increased hardness, with PLA containing 10 wt.% of date pits showing the highest hardness with an increase of 20% compared with pure PLA. While the enhancement due to 5 h of annealing recorded the highest hardness value that, reached 8% higher than pure PLA.

Table 3 and Table 4 presented the Young’s modulus and ultimate compressive strength of the PLA samples after being subjected to different heat treatment durations. As expected, the results demonstrate that the compression properties of the PLA have been enhanced. The best performance was shown after 5 h of heat treatment, which reached 1541 MPa and 60.22 MPa, improving by 2.7% and 25.7%, respectively. The decrease in mechanical properties observed after heat treatment for more than 5 h can be attributed to the initiation of PLA degradation.

**Figure 5:**
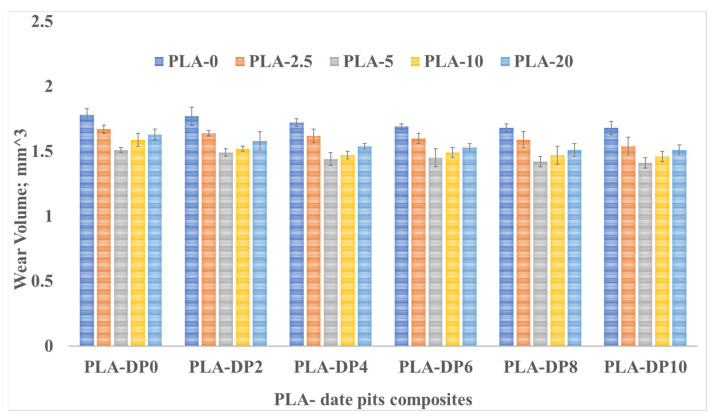
Wear volume of PLA–DP composites with different DP weight fractions and heat treatment duration time.

**Figure 6:**
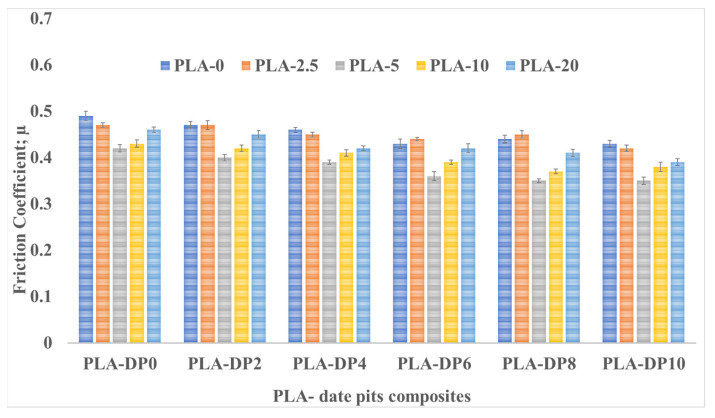
The friction coefficient of PLA–DP composites with different DP weight fractions and heat treatment duration time.

To assess the wear resistance of the PLA composites, the wear volume and friction coefficient of the neat PLA and PLA/date pits composites at various applied normal loads were measured, and the results are displayed in Figure 5 and Figure 6, respectively. As the concentration of date pits increased, the wear volume decreased, which can be attributed to the improved mechanical properties of the composites. This indicates that the strength of the PLA composites increased with a greater concentration of date pits particles. As a result, the wear resistance of the PLA composites was improved due to the strong bonding between the PLA matrix and the date pits, which restrained the composite surface degradation during the friction test. The wear volume of untreated PLA was reduced by 6% when the weight fraction of date pits was increased from 2 to 10 wt.%, as shown in Figure 4.

Moreover, the figure indicates that heat-treated PLA/date pits composites exhibited a wear reduction of 25.5%. Conversely, a significant increase in the wear volume was observed with an increase in the applied normal load, which could be attributed to the heat resulting from the kinetic energy generated by the relative motion between the rubbing surfaces. This heat softened the surface of the contact zone of the PLA composite and created high shear resistance, leading to a considerable increase in the wear volume. Figure 4 and Figure 5 also illustrate the change in the wear volume and friction coefficient with the change in the sliding distance. Although increasing the sliding time generally led to an increase in the wear volume, the incorporation of date pits into the composite materials still reduced the wear volume.

**Figure 7:**
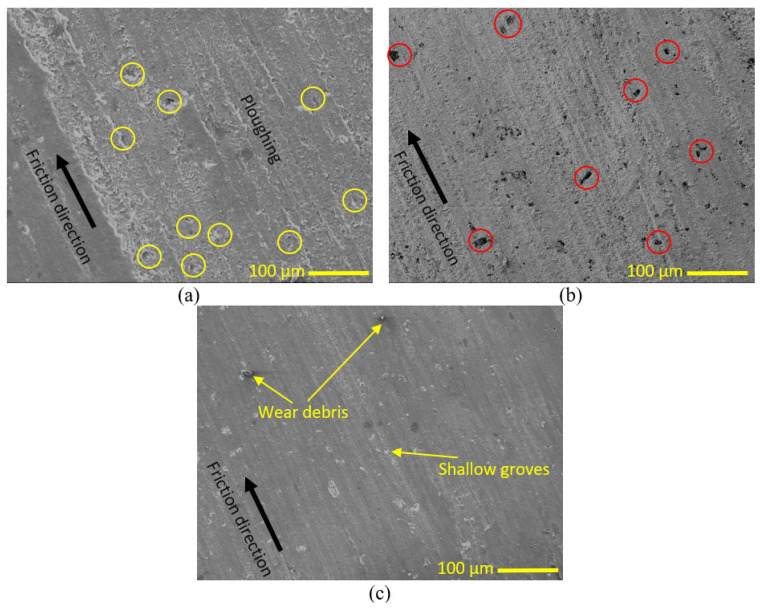
SEM of pure PLA (**a**) without heat treatment, (**b**) after 2.5 h, and (**c**) after 5 h.

For a better understanding of the wear characteristics of PLA samples before and after the heat treatment process, an analysis of their worn surfaces was carried out using SEM, as depicted in Figure 7. According to Figure 7a, the pure PLA-worn surface demonstrated plowing as a result of abrasive wear. During the friction test, microvoids on the surface of the pure PLA sample became deformed. However, the surface weakness caused the worn surface to degrade, leading to a significant wear volume increase. Yellow circles highlight the remaining microvoids on the surface of the sample. The surface of the PLA sample after 2.5 h of heat treatment is illustrated in Figure 7b, where a reduction in plowing can be observed. As previously mentioned, after 2.5 h of exposure to heat, numerous voids coalesced and formed larger voids. This can be seen in the red circle, where the surface deformation during the friction test could not overcome the large voids, resulting in their persistence. Figure 7c displays the surface of the PLA sample after 5 h of heat treatment, which appears smooth without any voids and with only a small amount of debris on its surface. The improvement in the surface of the PLA sample after 5 h of heat treatment can be attributed to the increase in the sample’s strength. As a result, the wear volume of the sample decreased significantly.

**Figure 8:**
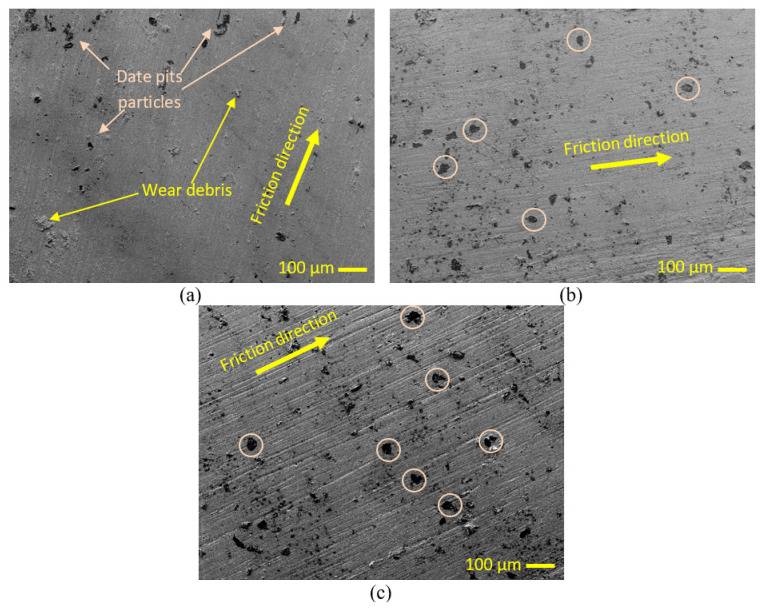
SEM of PLA composites (**a**) 2.5 wt.%(DP), (**b**) 6 wt.%(DP), and (**c**) 10 wt.%(DP).

After 5 h of heat treatment, Figure 8a displays the PLA/date pit worn surface containing 2 wt.% of date pits. The image reveals the presence of date particles and debris on the sample’s surface. The sample’s surface remains smooth, like the surface of pure PLA in Figure 8c. Figure 8b depicts the composite sample containing 6 wt.% of date pits, where an increase in the weight fraction of the pits has led to a rise in the number of date particles on the surface. Pink circles in the image highlight the presence of some agglomerations of date pit particles on the surface of the composite sample. The worn surface of the composite sample with a weight fraction of 10% is presented in Figure 8c. The image reveals a noticeable increase in the number of date pit particles on the surface and an increase in agglomeration. Moreover, the surface morphology has become rougher in the direction of friction due to the presence of scraped date pit particles.

**Figure 9:**
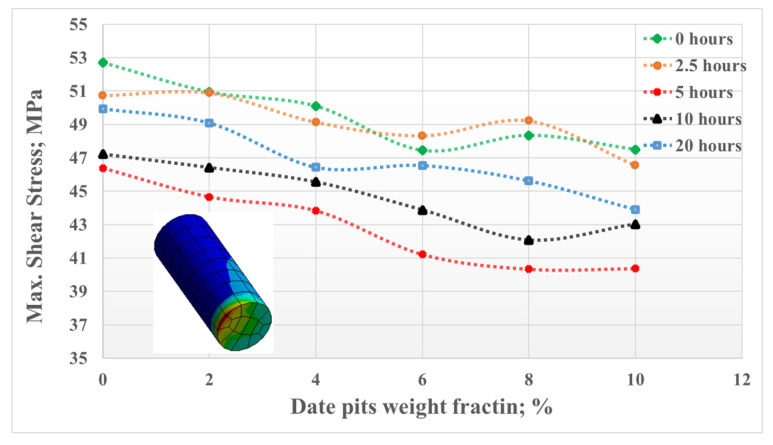
Maximum shear stress on the PLA/DP sample surfaces, frictional model.

The load-carrying capacity of the material can be assessed by measuring stress distribution across the prepared composite samples. The finite element analysis findings support that the inclusion of date pits particles in the PLA, along with a 5 h heat treatment, enhances their load-carrying capacity. Figure 9 illustrates the maximum shear stresses that occurred on the surfaces of the samples during the friction test under a normal load of 30 N. The results obtained from the test mirror the performance observed in the knee joint model. The finite element analysis results of the two models are consistent with the experimental mechanical and tribological results.

**Figure 10:**
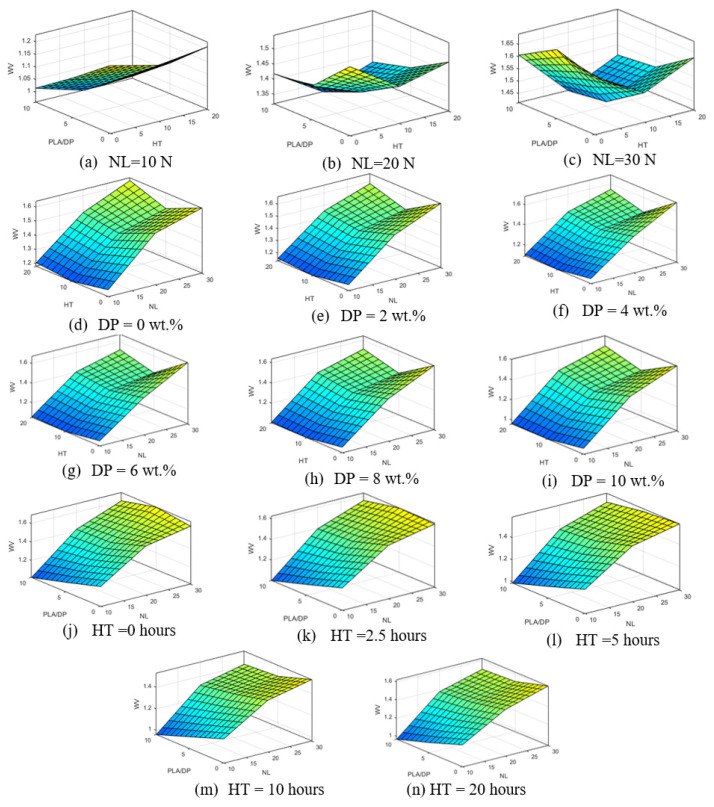
Wear volume in relation to change of normal load (**a**–**c**), DP weight fraction (**d**–**i**), heat treatment time (**j**–**n**), and sliding distance of 360 m.

The ANFIS model was employed to obtain the wear volume of PLA and PLA/DP composites based on the first three input variables, the normal load (NL), date pits weight fraction (PLA/DP), and heat treatment duration time (HT). The impact of the three parameters on the output variation of wear volume is presented in Figure 10. Figure 10 indicates that the normal load plays a significant role in the wear volume and is responsible for altering the surface contour of the wear volume. Generally, increasing the normal load results in a corresponding increase in the wear volume. However, variations in the date pits’ weight fraction and heat treatment duration time have a minor impact on the surface contour of the wear volume. Notably, an increase in the date pits weight fraction leads to a reduction in the wear volume, a finding consistent with experimental results. It is worth noting that neither the alteration of the date pits weight fraction nor the applied normal load changes the fact that 5 h of heat treatment corresponds to the minimum wear volume.

**Figure 11:**
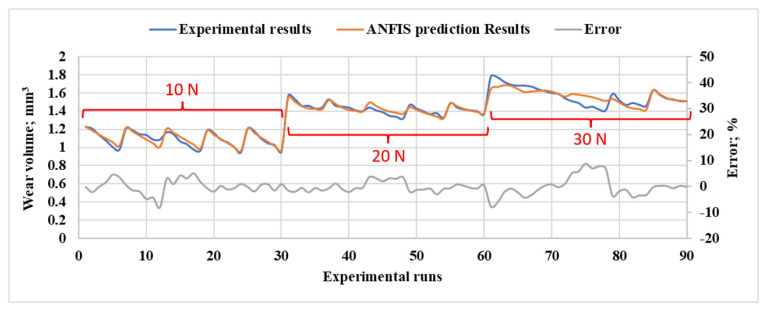
The comparison between experimental measured wear rate and ANFIS predicted ones for different normal loads, DP wt.%, heat treatment time, and 360 m sliding distance.

In Figure 11, a comparison is presented between the experimentally measured wear volume and the corresponding wear volume predicted by ANFIS. The points that exhibit a high degree of similarity with the experimental data are the points used for the training process. Other points represent the test data, which have a noticeable difference from the experimental data. However, the average percentage error between the predicted and experimental data is less than 9.31 × 10^−3^%, indicating that the ANFIS model provides a reasonably accurate prediction of the wear volume.

**Figure 12:**
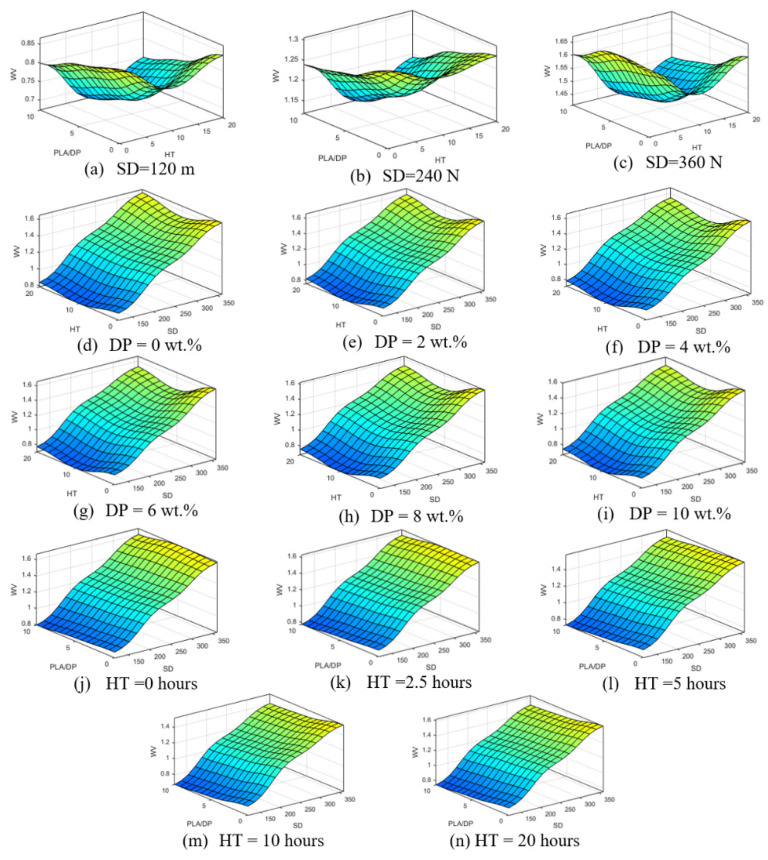
Wear volume in relation to change of sliding distance (**a**–**c**), DP weight fraction (**d**–**i**), heat treatment time (**j**–**n**), and normal load 30 N.

Figure 12 presents the influence of sliding distance (SD), date pits weight fraction (PLA/DP), and heat treatment duration time (HT) on the wear volume. The results show that altering the sliding distance leads to a change in the surface contour of the wear volume. Similar to the previous model that included normal load, the variations in the date pits weight fraction and heat treatment duration time only have a minor impact on the surface contour of the wear volume.

**Figure 13:**
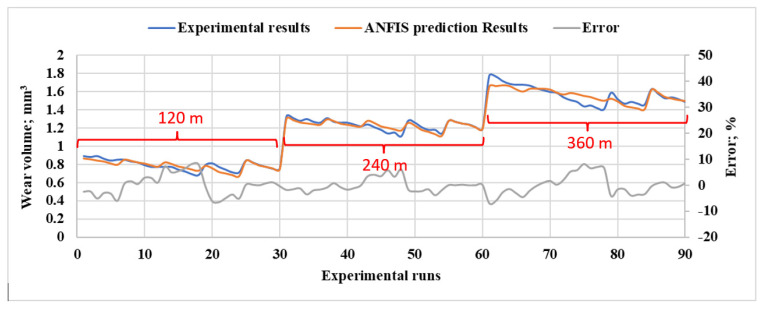
The comparison between experimental measured wear rate and ANFIS predicted rates for different sliding distances, DP wt.%, heat treatment time, and 30 N normal load.

Figure 13 shows a comparison between the experimentally measured wear volume and the wear volume predicted by the ANFIS model. Similar to the previous model, the points used for training the model exhibit high similarity with the experimental data. Other points represent the test data, which displays a noticeable difference from the experimental data. Nonetheless, the average percentage error between the predicted and experimental data is less than 4.4 × 10^−2^%.

## 4. Conclusions

Estimating wear volume for composite materials is complex and non-linear. Artificial intelligence approaches and expert systems like ANFIS are designed for modeling non-linear systems. Here are the investigation conclusions:

▪ ANFIS-based estimation models are proposed to predict the wear volume of PLA green composites. The models consider weight fractions of date pits, heat treatment durations, loads, and sliding distances. Experimental tribology testing provided the data for model training.
▪ A comprehensive production process of the PLA green composite was conducted, including heat treatment.
▪ The mechanical and tribological properties of PLA composites were determined through experimental testing.
▪ Finite element models were used to assess the load-carrying capacity of the composites.
▪ Incorporating 10% date pit particles increased hardness, Young’s modulus, and ultimate compressive strength.
▪ Heat treatment enhanced mechanical properties and reduced wear volume, improving tribological properties.
▪ Finite element analysis showed a decrease in contact stresses with date pit particle incorporation and heat treatment.
▪ The ANFIS model accurately predicted wear volume under different conditions. The model had an average percentage error of less than 9.31 × 10^−3^%.

## Figures and Tables

**Table 1:** Sample designation (**a**) DP weight fraction-based, and (**b**) annealing time.

(a)		(b)	
Sample Code	DP (wt.%)	Sample Code	Hours
PLA-DP0	0	PLA-0	0
PLA-DP2	2	PLA-2.5	2.5
PLA-DP4	4	PLA-5	5
PLA-DP6	6	PLA-10	10
PLA-DP8	8	PLA-20	20
PLA-DP10	10		

**Table 2:** Hardness (D-Index) of PLA composite with different weight fractions of date pits at different heat treatment duration times.

	PLA-0	PLA-2.5	PLA-5	PLA-10	PLA-20
PLA-DP0	79.5 ± 0.3	81.1 ± 0.7	86 ± 0.6	83.6 ± 0.8	85.4 ± 0.6
PLA-DP2	91 ± 0.6	88.9 ± 0.8	93 ± 0.4	92.2 ± 0.5	91.6 ± 0.8
PLA-DP4	92.6 ± 0.4	91.4 ± 0.8	94.2 ± 0.7	93.6 ± 0.6	92.7 ± 0.7
PLA-DP6	93.6 ± 0.7	93.4 ± 0.5	95.4 ± 0.8	94.7 ± 0.8	94.1 ± 0.4
PLA-DP8	93.8 ± 0.8	92.8 ± 0.4	95.7 ± 0.8	93.8 ± 0.7	94.2 ± 0.5
PLA-DP10	95 ± 0.5	95.3 ± 0.6	96.7 ± 0.5	94.8 ± 0.4	95.4 ± 0.8

**Table 3:** Young’s modulus in (MPa) of PLA composite with different weight fractions of date pits at different heat treatment duration times.

	PLA-0	PLA-2.5	PLA-5	PLA-10	PLA-20
PLA-DP0	1501 ± 6.3	1227 ± 8.4	1479 ± 10.2	1394 ± 12.4	1435 ± 9.4
PLA-DP2	1613 ± 10.1	1520 ± 6.6	1630 ± 8.5	1580 ± 9.6	1533 ± 12.2
PLA-DP4	1724 ± 8.6	1658 ± 7.8	1760 ± 12.1	1640 ± 12.5	1610 ± 8.3
PLA-DP6	1839 ± 15.1	1805 ± 10.3	1873 ± 9.3	1863 ± 10.2	1835 ± 8.7
PLA-DP8	1844 ± 10.3	1790 ± 12.5	1880 ± 10.4	1810 ± 8.4	1795 ± 9.1
PLA-DP10	1944 ± 13.2	1868 ± 9.1	2064 ± 12.3	1951 ± 9.6	1923 ± 10.3

**Table 4:** Ultimate compressive strength in (MPa) of PLA composite with different weight fractions of date pits at different heat treatment duration times.

	PLA-0	PLA-2.5	PLA-5	PLA-10	PLA-20
PLA-DP0	47.9 ± 0.5	53.4 ± 0.6	60.22 ± 0.7	56.82 ± 0.5	57.37 ± 0.7
PLA-DP2	48.94 ± 0.3	56.61 ± 0.8	60.87 ± 0.9	58.55 ± 0.7	57.52 ± 0.5
PLA-DP4	51.52 ± 0.7	55.53 ± 0.4	61.23 ± 0.8	60.14 ± 0.5	60.24 ± 0.7
PLA-DP6	53.41 ± 0.4	57.11 ± 0.5	61.88 ± 0.4	59.55 ± 0.8	58.16 ± 0.8
PLA-DP8	57.52 ± 0.8	59.45 ± 0.7	63.11 ± 0.8	61.27 ± 0.6	60.71 ± 0.4
PLA-DP10	59.99 ± 0.4	64.25 ± 0.6	68.21 ± 0.5	65.99 ± 0.5	65.15 ± 0.5

## Data Availability

Not applicable.

## References

[1] Gross J.-B., Guillaume C., Gegout-Pottie P., Mainard D., Presle N. (2014). Synovial fluid levels of adipokines in osteoarthritis: Association with local factors of inflammation and cartilage maintenance. Biomed. Mater. Eng..

[2] Espallargas N., Fischer A., Muñoz A.I., Mischler S., Wimmer M.A. (2017). In-situ generated tribomaterial in metal/metal contacts: Current understanding and future implications for implants. Biotribology.

[3] Smith A.J., Dieppe P., Vernon K., Porter M., Blom A.W. (2012). Failure rates of stemmed metal-on-metal hip replacements: Analysis of data from the National Joint Registry of England and Wales. Lancet.

[4] Zhao W. (2023). Progress of stem cell research in knee osteoarthritis. Highlights Sci. Eng. Technol..

[5] Hussain O., Ahmad B., Saleem S.S. (2023). Biomaterials for artificial knee joint replacement: A review. Int. J. Mater. Eng. Innov..

[6] Kurtz S.M., Ong K.L., Lau E., Bozic K.J. (2014). Impact of the economic downturn on total joint replacement demand in the United States: Updated projections to 2021. JBJS.

[7] Mai K., Verioti C., Ezzet K.A., Copp S.N., Walker R.H., Colwell C.W. (2010). Incidence of ‘squeaking’after ceramic-on-ceramic total hip arthroplasty. Clin. Orthop. Relat. Res..

[8] Ji R., Qi Z., Chen J., Zhang L., Lin K., Lu S., Li Y. (2023). Numerical and experimental investigation on the abrasive flow machining of artificial knee joint surface. Crystals.

[9] Pani R., Behera R.R., Roy S. (2023). Corrosion Behaviour of Metallic Biomaterials in Physiological Environments. Handbook of Research on Corrosion Sciences and Engineering.

[10] Fouly A., Assaifan K.A., Alnaser I.A., Hussein O.A., Abdo H.S. (2022). Evaluating the mechanical and tribological properties of 3D printed polylactic-acid (PLA) green-composite for artificial implant: Hip joint case study. Polymers.

[11] Rao A.J., Gibon E., Ma T., Yao Z., Smith R.L., Goodman S.B. (2012). Revision joint replacement, wear particles, and macrophage polarization. Acta Biomater..

[12] Enab T.A. (2012). A comparative study of the performance of metallic and FGM tibia tray components in total knee replacement joints. Comput. Mater. Sci..

[13] Zietz C., Bergschmidt P., Lange R., Mittelmeier W., Bader R. (2013). Third-body abrasive wear of tibial polyethylene inserts combined with metallic and ceramic femoral components in a knee simulator study. Int. J. Artif. Organs.

[14] Fouly A., Abdo H.S., Seikh A.H., Alluhydan K., Alkhammash H.I., Alnaser I.A., Abdo M.S. (2021). Evaluation of Mechanical and Tribological Properties of Corn Cob-Reinforced Epoxy-Based Composites—Theoretical and Experimental Study. Polymers.

[15] Fouly A., Nasr M.N., Fath El Bab A.M., Abouelsoud A.A. (2015). Design and modeling of micro tactile sensor with three contact tips for self-compensation of contact error in soft tissue elasticity measurement. IEEJ Trans. Electr. Electron. Eng..

[16] Khalil H.A., Yahya E.B., Jummaat F., Adnan A.S., Olaiya N.G., Rizal S., Abdullah C.K., Pasquini D., Thomas S. (2022). Biopolymers based aerogels: A review on revolutionary solutions for smart therapeutics delivery. Prog. Mater. Sci..

[17] Ahmed F., Alnaser I.A., Assaifan A.K., Abdo H.S. (2023). Developing PMMA/coffee husk green composites to meet the individual requirements of people with disabilities: Hip spacer case study. J. Funct. Biomater..

[18] Premkumar J., SonicaSree K., Sudhakar T. (2021). Polymers in Biomedical Use. Handbook of Polymer and Ceramic Nanotechnology.

[19] Jacobs J.J., Roebuck K.A., Archibeck M., Hallab N.J., Glant T.T. (2001). Osteolysis: Basic science. Clin. Orthop. Relat. Res..

[20] Galliera E., Ragone V., Marazzi M.G., Selmin F., Banci L., Romanelli M.M.C. (2018). Vitamin E-stabilized UHMWPE: Biological response on human osteoblasts to wear debris. Clin. Chim. Acta.

[21] Fouly A., Nabhan A., Badran A. (2022). Mechanical and Tribological Characteristics of PMMA Reinforced by Natural Materials. Egypt. J. Chem..

[22] Zeman J., Ranuša M., Vrbka M., Gallo J., Křupka I., Hartl M. (2018). UHMWPE acetabular cup creep deformation during the run-in phase of THA’s life cycle. J. Mech. Behav. Biomed. Mater..

[23] Bakhtiar S.M., Butt H.A., Zeb S., Quddusi D.M., Gul S., Dilshad E. (2018). 3D printing technologies and their applications in biomedical science. Omics Technologies and Bio-Engineering.

[24] Bozkurt Y., Karayel E. (2021). 3D printing technology; methods, biomedical applications, future opportunities and trends. J. Mater. Res. Technol..

[25] Subramaniam S.R., Samykano M., Selvamani S.K., Ngui W.K., Kadirgama K., Sudhakar K., Idris M.S. (2019). 3D printing: Overview of PLA progress. AIP Conf. Proc..

[26] Radenkovic D., Solouk A., Seifalian A. (2016). Personalized development of human organs using 3D printing technology. Med. Hypotheses.

[27] Fernandes J., Deus A.M., Reis L., Vaz M.F., Leite M. Study of the influence of 3D printing parameters on the mechanical properties of PLA. Proceedings of the 3rd International Conference on Progress in Additive Manufacturing (Pro-AM 2018).

[28] Zhang J., Hori N., Takemura A. (2023). Effect of natural biomass fillers on the stability, degradability, and elasticity of crop straws liquefied polyols-based polyurethane foams. J. Appl. Polym. Sci..

[29] Liu Z., Lei Q., Xing S. (2019). Mechanical characteristics of wood, ceramic, metal and carbon fiber-based PLA composites fabricated by FDM. J. Mater. Res. Technol..

[30] Fouly A., Alnaser I.A., Assaifan A.K., Abdo H.S. (2022). Evaluating the Performance of 3D-Printed PLA Reinforced with Date Pit Particles for Its Suitability as an Acetabular Liner in Artificial Hip Joints. Polymers.

[31] Leite M., Varanda A., Ribeiro A.R., Silva A., Vaz M.F. (2018). Mechanical properties and water absorption of surface modified ABS 3D printed by fused deposition modelling. Rapid Prototyp. J..

[32] Bhandari S., Lopez-Anido R.A., Gardner D.J. (2019). Enhancing the interlayer tensile strength of 3D printed short carbon fiber reinforced PETG and PLA composites via annealing. Addit. Manuf..

[33] Srithep Y., Nealey P., Turng L.-S. (2013). Effects of annealing time and temperature on the crystallinity and heat resistance behavior of injection-molded poly (lactic acid). Polym. Eng. Sci..

[34] Jayanth N., Jaswanthraj K., Sandeep S., Mallaya N.H., Siddharth S.R. (2021). Effect of heat treatment on mechanical properties of 3D printed PLA. J. Mech. Behav. Biomed. Mater..

[35] Atia M.G., Salah O. Fuzzy logic with load compensation for upper limb exoskeleton control based on IMU data fusion. Proceedings of the 2018 IEEE International Conference on Robotics and Biomimetics (ROBIO).

[36] Rajpurohit S.R., Dave H.K. (2020). Prediction and optimization of tensile strength in FDM based 3D printing using ANFIS. Optimization of Manufacturing Processes.

[37] Sahin Y. (2005). The prediction of wear resistance model for the metal matrix composites. Wear.

[38] Vijayakumar S., Karunamoorthy L. (2012). Modelling wear behaviour of Al–SiC metal matrix composites: Soft computing technique. Tribol.-Mater. Surf. Interfaces.

[39] Asker A., Salah O., El-Bab A.M.F., Ramadan A.A., Assal S.M., Sessa S., Abo-Ismail A. ANFIS based jacobian for a parallel manipulator mobility assistive device. Proceedings of the 2014 UKACC International Conference on Control (CONTROL).

[40] Chhabra D., Deswal S., Kaushik A., Garg R.K., Kovács A., Khargotra R., Singh T. (2022). Analysis of fused filament fabrication parameters for sliding wear performance of carbon reinforced polyamide composite material fabricated parts using a hybrid heuristic tool. Polym. Test..

[41] Ibrahim M.A., Şahin Y., Ibrahim A., Gidado A.Y., Yahya M.N. (2021). Specific Wear Rate Modeling of Polytetraflouroethylene Composites Via Artificial Neural Network (ANN) and Adaptive Neuro Fuzzy Inference System (ANFIS) Tools. Virtual Assistant.

[42] Ahmad A., Imtiaz H. (2019). Chemical composition of date pits: Potential to extract and characterize the lipid fraction. Sustainable Agriculture Reviews 34.

[43] Zhao H., Allanson D., Ren X.J. (2015). Use of shore hardness tests for in-process properties estimation/monitoring of silicone rubbers. J. Mater. Sci. Chem. Eng..

[44] (1999). Fibre-Reinforced Plastic Composites—Determination of Compressive Properties in the In-Plane Direction.

[45] (2008). Standard Test Method for Wear Testing with a Pin-on-Disk Apparatus.

[46] Kuminek T., Aniołek K., Młyńczak J. (2015). A numerical analysis of the contact stress distribution and physical modelling of abrasive wear in the tram wheel-frog system. Wear.

